# Hydrops Fetalis and Persistent Pulmonary Hypertension in a Neonate with Anti-E Alloimmunization

**DOI:** 10.1155/2019/3736870

**Published:** 2019-03-04

**Authors:** Amit Sharma, Pournima Navalkele, Jagdish Desai, Prashant Agarwal

**Affiliations:** ^1^Department of Neonatal-Perinatal, Children's Hospital of Michigan, Neonatal-Perinatal Medicine, Detroit, MI, USA; ^2^Department of Pediatrics, SSM Health Cardinal Glennon Children's Hospital, Hematology/Oncology, St. Louis, MO, USA; ^3^Department of Neonatal-Perinatal, University of Mississippi Medical Center, Neonatology, Jackson, MS, USA

## Abstract

Anti-E alloimmunization is the third most common cause of neonatal hemolytic disease, typically causing mild to moderate hemolytic anemia. We report an unusual case of severe hydrops fetalis and persistent pulmonary hypertension (PPHN) in a neonate with anti-E alloimmunization. Our case emphasizes the importance of close surveillance for development of severe fetal hemolytic anemia and possible need for antenatal intervention. These neonates may also need vigilant monitoring for PPHN.

## 1. Introduction

Anti-E alloimmunization is detected in 14–20% of pregnant women [1]. In the period of Rhesus (Rh) immunization, anti-E alloimmunization is becoming a significant cause of immune mediated hydrops fetalis [2]. Anti-E alloimmunization has been reported to cause mild to moderate hemolytic anemia, but rarely associated with severe anemia in the fetus [3–5]. In one series of 210 fetuses transfused over an 11-year period, only one fetus affected by maternal anti-E antibody required intrauterine transfusion [6].

## 2. Case

A late preterm (36 5/7 weeks) male neonate was born at an outside facility to a 29-year-old, Caucasian, Gravida 5 Para 5 mother needing cesarean section for category II fetal heart rate tracing. Mother had limited prenatal care. Antenatal ultrasound (US) during second trimester was normal. Maternal TORCH, Parvovirus B 19, and Epstein Barr Virus workup was negative. She had no family history of anemia, gall bladder surgery, splenectomy, or hydrops fetalis. All her older children were born healthy except one, who required phototherapy at birth for hyperbilirubinemia.

At birth, the newborn was appropriate for gestational age (birth weight 2600 g) with Apgar score of 4, 6, and 7 at 1, 5, and 10 minutes, respectively. He was intubated for poor respiratory effort and was admitted to neonatal intensive care unit (NICU). On examination, he appeared pale and noticed to have firm, distended abdomen with hepatosplenomegaly and generalized body wall edema. There was no cephalhematoma, subgaleal bleed, or bruising. Cord blood analysis showed severe congenital anemia with hematocrit of 20.3% and packed red blood cells (PRBC) were transfused. Infant's blood group was A+ with direct coombs 2+ for anti-IgG with anti-E found on elution. Mother's blood group was A+, but antibody screen was unknown at birth hospital.

Infant was placed on a mechanical ventilator and initial chest radiograph revealed cardiomegaly. Fresh frozen plasma and platelets were also transfused for active bleeding from the umbilical stump. Diagnosis of hydrops fetalis was confirmed by echocardiogram showing a structurally normal heart with small pericardial effusion, ascites on abdominal US, and generalized edema on exam.

Laboratory workup showed white blood cell count 10,400/ml, hemoglobin 9.1 g/dl, and hematocrit 25.1%; reticulocyte count was 18%, lactate dehydrogenase (LDH)>4000 u/l, and platelets 154,000/ml; and peripheral smear showed numerous nucleated red blood cells with moderate schistocytes, target cell, and few burr cells. Total bilirubin at 3 hours of life was 4.6 mg/dl.

Infant was transferred to our level IV NICU for further management due to hypoxemic respiratory failure. He continued to have labile oxygen saturation despite being on 100% oxygen, for which inhaled nitric oxide (iNO) was started at 20ppm and repeat echocardiogram showed features of persistent pulmonary hypertension of newborn (PPHN) including suprasystemic right ventricular pressure with severe tricuspid valve insufficiency. He became hypotensive requiring multiple vasopressor support.

Partial exchange transfusion was deferred due to hemodynamic instability and PRBC were transfused in small aliquots for severe anemia. At 12 hours of life, he was started on phototherapy for total bilirubin of 13 mg/dl.

Although anti-E hemolytic disease rarely leads to severe hydrops, consultant hematologist agreed with the suspected diagnosis due to evidence of high reticulocyte count (18.1%), LDH >4000 mg/dL, numerous nucleated red blood cells, and normoblasts on peripheral smear (transfused specimen) along with positive direct coombs test findings. Maternal blood group antibody screen and placental pathology remained unavailable from the birth hospital. Cord blood ferritin was normal ruling out chronic fetomaternal blood loss. Alpha thalassemia was ruled out as initial mean corpuscular volume was high and there was no evidence of many target cells on peripheral smear. Congenital aplastic or dyserythropoietic anemia was ruled out due to initial high reticulocyte count. Infectious etiology was ruled out due to negative herpes simplex virus 1 and 2 polymerase chain reaction (PCR), cytomegalovirus (CMV) PCR, urine CMV culture, and parvovirus PCR. Baby was seropositive for Epstein Barr Virus, possibly from maternal antibody.

He continued to remain hypoxemic despite maximal ventilatory support and inhaled nitric oxide, so eventually placed on extracorporeal membrane oxygenation (ECMO). Head US prior to initiating ECMO was normal. Repeat head US on day 2 of life while on ECMO showed development of large left intracranial hemorrhage (ICH) with midline shift. ECMO was promptly discontinued and the patient was placed back on mechanical ventilation. Parents were counselled about poor prognosis in view of hypoxemic respiratory failure and large ICH. Parents agreed for comfort measures. Soon after withdrawal of life support, the patient passed away. Autopsy report showed extensive extramedullary hematopoiesis within multiple organs including liver and spleen.

## 3. Discussion

Traditionally, a high level of suspicion and early recognition of anti-E alloimmunization was required, as there is poor correlation between maternal anti-E titers, cord blood hemoglobin and severity of hemolytic disease especially in the subsequent pregnancy [3, 7].

In 2018, American College of Obstetrician and Gynecologists published guidelines suggesting that an antibody titer between 1:8 and 1:32 is considered critical and associated with a significant risk for severe erythroblastosis fetalis and hydrops [8].

Amniocentesis has been supplanted by serial middle cerebral artery (MCA) Dopplers in the management of the red cell alloimmunized pregnancy. Mari et al. have shown that a cut-off of 1.5 multiples of median (MoM) on Doppler peak systolic velocity (PSV) in the MCA has 100% sensitivity in the prediction of moderate to severe fetal anemia [9]. The PSV in the MCA is higher in fetuses with anemia whereas it decreases when the fetal hematocrit rises [10]. Moran et al. revealed that using MCA PSV, fetal anemia is treatable with 90% favorable outcome before hydrops happens [11].

In our patient, unfortunately, limited prenatal care led to postnatal diagnosis of severe congenital hemolytic anemia. Subsequently, the striking finding of PPHN with eventual fatal outcome warrants discussion here.

PPHN is a result of persistence of high pulmonary vascular resistance after birth. The common perinatal risk factors associated with development of PPHN are perinatal hypoxemia, meconium aspiration syndrome, diaphragmatic hernia, neonatal sepsis, and pneumonia [12]. Maternal risk factors associated with PPHN include diabetes mellitus, obesity, and medication such as aspirin, nonsteroidal anti-inflammatory agents, and serotonin reuptake inhibitors agents [13, 14].

A few case reports have described the association of congenital anemia with development of PPHN [15, 16]. Congenital anemia leads to systemic hypoxemia, which in turn may exacerbate pulmonary vascular resistance [17]. Reduced hemoglobin decreases systemic transport of oxygen, thus causing hypoxemia, which is a known risk factor for PPHN. Also, hemolytic anemia leads to dysregulated arginine metabolism and reduction in nitric oxide bioavailability, a potent vasodilator for pulmonary circulation [18, 19].

In our patient, as PPHN was not related to the known perinatal or maternal risk factors, the severe congenital anemia may have played a role in the development of PPHN. Based on our literature review, this is the first case report associating severe congenital anti-E hemolytic anemia with the development of PPHN.

In conclusion, maternal anti-E alloimmunization should prompt a clinician to monitor prenatal antibody titers. Critical anti-E titers may need further investigations such as fetal MCA Doppler or amniocentesis in order to predict the severity of fetal hemolytic anemia. In the absence of known risk factors, severe congenital hemolytic anemia may necessitate vigilant monitoring for development of PPHN.
